# European Society of Breast Imaging (EUSOBI) guidelines on the management of axillary lymphadenopathy after COVID-19 vaccination: 2023 revision

**DOI:** 10.1186/s13244-023-01453-2

**Published:** 2023-07-19

**Authors:** Simone Schiaffino, Katja Pinker, Andrea Cozzi, Veronica Magni, Alexandra Athanasiou, Pascal A. T. Baltzer, Julia Camps Herrero, Paola Clauser, Eva M. Fallenberg, Gabor Forrai, Michael H. Fuchsjäger, Fiona J. Gilbert, Thomas Helbich, Fleur Kilburn-Toppin, Christiane K. Kuhl, Mihai Lesaru, Ritse M. Mann, Pietro Panizza, Federica Pediconi, Francesco Sardanelli, Tamar Sella, Isabelle Thomassin-Naggara, Sophia Zackrisson, Ruud M. Pijnappel

**Affiliations:** 1 Imaging Institute of Southern Switzerland (IIMSI), Ente Ospedaliero Cantonale (EOC), Lugano, Switzerland; 2 Division of General and Paediatric Radiology, Department of Biomedical Imaging and Image-Guided Therapy, Medical University of Vienna, Vienna, Austria; 3 Department of Radiology, Breast Imaging Service, Memorial Sloan Kettering Cancer Center, New York, NY USA; 4 Department of Biomedical Sciences for Health, Università degli Studi di Milano, Milan, Italy; 5 Breast Imaging Department, MITERA Hospital, Athens, Greece; 6 Área de Salud de la Mama, Ribera Salud Grupo, Valencia, Spain; 7 Department of Diagnostic and Interventional Radiology, Klinikum Rechts Der Isar, Technical University of Munich (TUM), Munich, Germany; 8 Department of Radiology, Duna Medical Center, Budapest, Hungary; 9 Division of General Radiology, Department of Radiology, Medical University Graz, Graz, Austria; 10 Department of Radiology, University of Cambridge, Cambridge, UK; 11 University Hospital of Aachen, Rheinisch-Westfälische Technische Hochschule, Aachen, Germany; 12 Radiology and Imaging Laboratory, Fundeni Institute, Bucharest, Romania; 13 Department of Radiology, Radboud University Medical Centre, Nijmegen, The Netherlands; 14 The Netherlands Cancer Institute, Amsterdam, The Netherlands; 15 Breast Imaging Unit, IRCCS Ospedale San Raffaele, Milan, Italy; 16 Department of Radiological, Oncological and Pathological Sciences, Università degli Studi di Roma “La Sapienza”, Rome, Italy; 17 Unit of Radiology, IRCCS Policlinico San Donato, San Donato Milanese, Italy; 18 Department of Diagnostic Imaging, Hadassah Hebrew University Medical Center, Jerusalem, Israel; 19 Department of Radiology, Hôpital Tenon APHP, Sorbonne Université, Paris, France; 20 Diagnostic Radiology, Department of Translational Medicine, Skåne University Hospital, Lund University, Malmö, Sweden; 21 Department of Imaging, University Medical Centre Utrecht, Utrecht University, Utrecht, The Netherlands

**Keywords:** COVID-19 vaccines, Lymphadenopathy, Mammography, Ultrasonography (breast), Magnetic resonance imaging

## Abstract

Axillary lymphadenopathy is a common side effect of COVID-19 vaccination, leading to increased imaging-detected asymptomatic and symptomatic unilateral axillary lymphadenopathy. This has threatened to negatively impact the workflow of breast imaging services, leading to the release of ten recommendations by the European Society of Breast Imaging (EUSOBI) in August 2021. Considering the rapidly changing scenario and data scarcity, these initial recommendations kept a highly conservative approach. As of 2023, according to newly acquired evidence, EUSOBI proposes the following updates, in order to reduce unnecessary examinations and avoid delaying necessary examinations. First, recommendation n. 3 has been revised to state that breast examinations should not be delayed or rescheduled because of COVID-19 vaccination, as evidence from the first pandemic waves highlights how delayed or missed screening tests have a negative effect on breast cancer morbidity and mortality, and that there is a near-zero risk of subsequent malignant findings in asymptomatic patients who have unilateral lymphadenopathy and no suspicious breast findings. Second, recommendation n. 7 has been revised to simplify follow-up strategies: in patients without breast cancer history and no imaging findings suspicious for cancer, symptomatic and asymptomatic imaging-detected unilateral lymphadenopathy on the same side of recent COVID-19 vaccination (within 12 weeks) should be classified as a benign finding (BI-RADS 2) and no further work-up should be pursued. All other recommendations issued by EUSOBI in 2021 remain valid.

**Graphical abstract:**

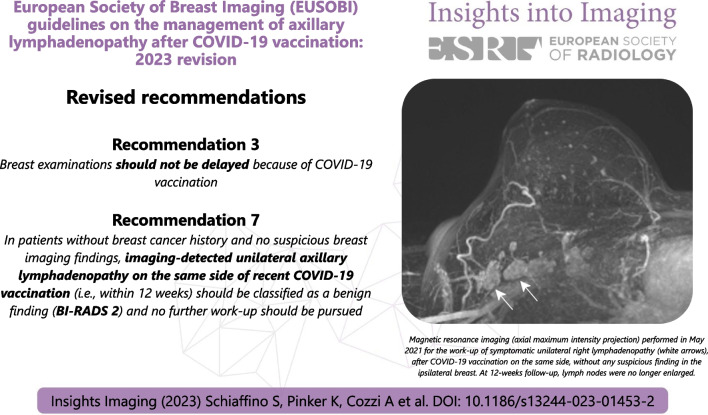

## Key points

- Worldwide COVID-19 vaccination campaigns have become commonplace.
- Post-vaccination COVID-19 lymphadenopathy has been reported in up to 44% of cases in at least one follow-up examination.
- Breast examinations should not be delayed because of COVID-19 vaccination.
- In average-risk patients without suspicious breast findings, ipsilateral imaging-detected axillary lymphadenopathy within 12 weeks after vaccination should be classified as BI-RADS 2.

## Background

Since the start of the COVID-19 vaccination campaigns in late December 2020, axillary lymphadenopathy quickly emerged as a common side effect [1], as confirmed by two studies published in 2022 [2, 3]. Moreover, as expected, compared with the incidence of symptomatic axillary lymphadenopathy reported in phase III vaccine trials [4, 5], there was an even higher incidence of imaging-detected asymptomatic and symptomatic unilateral axillary lymphadenopathy, up to 44% of patients who were followed up with breast imaging after vaccination in the largest published study to date which included 1217 patients [3] and up to 78% in a smaller study [6].

Considering the far-reaching impact of vaccination campaigns (as of June 2023, around 76% of the European population has received at least one dose) and the boosting recommendations that have been issued, this sudden and marked increase of a previously rare differential diagnosis in the management of unilateral axillary lymphadenopathy threatened to negatively impact the workflow of breast imaging services, especially in light of potential unnecessary additional imaging and invasive procedures. Thus, the need to quickly provide a solid consensus framework on the management of post-vaccination lymphadenopathy prompted the release, in August 2021, of ten recommendations by the European Society of Breast Imaging (EUSOBI) [7].

In acknowledgement of the rapidly changing scenario and the lack of data concerning some aspects (e.g. incidence and persistence of axillary lymphadenopathy, and the rates of vaccination-induced versus cancer-induced lymphadenopathy), these 2021 recommendations, as those published by the Society of Breast Imaging (SBI) [8], kept an overall highly conservative approach, even recommending a delay of screening mammograms around COVID-19 vaccinations. However, since then, predictive models developed based on data from the first pandemic waves revealed that delayed or missed screening tests have a negative effect on morbidity and mortality [9–11], and the proposed management framework of unilateral axillary lymphadenopathy has also allowed accurate discrimination between suspicious and non-suspicious cases. Indeed, current evidence points towards a near-zero risk of subsequent malignant findings in asymptomatic patients who have unilateral lymphadenopathy and no suspicious breast findings (no cancer cases in the largest published series to date of 407 women [3]). Consequently, the potential temporal overlap of COVID-19 vaccinations should no longer be considered a reason to reschedule periodic breast examinations, either in spontaneous or organized screening programs, prioritizing the restoration of normal screening and management of symptomatic patients and the appropriately timed follow-up of those with a previous diagnosis of breast cancer.

Likewise, sparse information on temporal changes and the eventual persistence of asymptomatic lymphadenopathy justified more stringent follow-up approaches among the first published recommendations and guidelines [7, 8, 12, 13]. The available data that shed light on these issues came from a longitudinal study on follow-up ultrasound of 88 asymptomatic patients with imaging-detected vaccine-associated axillary lymphadenopathy [2]. Among the 49 women who had follow-up ultrasound at a median of 12 weeks after vaccination, around half (51%, 25 patients) had persistent lymphadenopathy [2]. This supports a follow-up timeframe starting at 12 weeks or later, as proposed by the EUSOBI 2021 recommendations [7] and then endorsed by the revised SBI guidelines [14], compared with the 4–12 weeks option proposed by the first version of the SBI guidelines [8] and by the Radiology Scientific Expert Panel [12]. However, data from this study also keep open the discussion about potential further lengthening, considering that almost half of the patients still had lymphadenopathy at the 12-week follow-up.

Moreover, potential further persistence of lymphadenopathy could be enhanced by the simultaneous or staggered application of other vaccines such as influenza or shingles.

Therefore, as already done by the SBI [14], the purpose of this paper is to review the ten recommendations published in 2021 in light of progressively acquired evidence, identifying and updating those in which a conservative approach might affect the clinical routine of breast imaging services.

## Revised recommendations

Eight of the ten recommendations provided in August 2021 are still valid today, while two (recommendations n. 3 and n. 7) need to be revised according to new information. All recommendations are again presented below, and a brief discussion is proposed for those that have been revised.

1. In patients with previous history of breast cancer, vaccine injection (both doses for two-doses vaccines) should be performed in the contralateral arm or in the anterolateral thigh.

*This recommendation has not been revised.*

2. COVID-19 vaccination data (vaccination status, date, dose, injection site) of all patients presenting for breast imaging with any modality should be collected and made available to radiologists, including the cases of breast imaging performed for cancer staging and of follow-up imaging examinations.

*This recommendation has not been revised.*

3. *Revised as*:

Breast examinations should not be delayed because of COVID-19 vaccination.

*The previous recommendation was in favour of rescheduling breast examinations (before the vaccination or at least 12 weeks after the last injection). However, current evidence points towards a near-zero risk of subsequent malignant findings in asymptomatic patients who have unilateral lymphadenopathy and no suspicious breast findings (no cancer cases in the largest series published to date *[3]*). Moreover, predictive models developed based on data from the first pandemic waves revealed that delayed or missed screening tests have a negative effect on morbidity and mortality *[9–11]*. Therefore, the potential temporal overlap of COVID-19 vaccinations in combination or staggered with other vaccines known to cause transient lymphadenopathy should no longer be considered a reason to reschedule periodic breast examinations, either in spontaneous or organized screening programs*.

4. In patients newly diagnosed with breast cancer, all necessary breast imaging examinations with any modality must be performed without any delay due to vaccination, taking into consideration the risk of false positive lymph node findings.

*This recommendation has not been revised*.

5. The contralateral axilla and both breasts should be clinically examined using appropriate imaging to exclude malignancy in all patients with axillary symptoms and in all cases of imaging-detected unilateral axillary lymphadenopathy before vaccination or at least 12 weeks after.

*Aside from isolated reports of vaccine-associated lymphadenopathy up to 43 weeks after vaccination *[3]*, the available literature shows that most cases of vaccine-associated lymphadenopathy arise within 50 days from vaccination *[3]*. Thus, the 12-week follow-up threshold after vaccination—after which axillary lymphadenopathy should also prompt the examination of both breasts and of the contralateral axilla—is still considered appropriate and the recommendation has not been revised*.

6. In patients with or without previous breast cancer history, imaging-detected suspicious axillary lymphadenopathy contralateral to the vaccination side should be managed according to standard work-up protocols, including, when necessary, tissue sampling.

*This recommendation has not been revised*.

7. *Revised as*:

In patients without breast cancer history and no suspicious breast imaging findings, imaging-detected unilateral axillary lymphadenopathy on the same side of recent COVID-19 vaccination (i.e. within 12 weeks) should be classified as a benign finding (BI-RADS 2) and no further work-up should be pursued.

*In the previous version, the conservative approach adopted in the absence of sufficient available evidence led to different recommendations for patients with and without axillary symptoms. Today, this type of attitude is hardly justified and recommendation 7 has been simplified.*

8. In patients without breast cancer history, incidental unilateral axillary lymphadenopathy after COVID-19 vaccination coupled with ipsilateral suspicious findings in the breast at any imaging modality should be managed according to clinical practice, including biopsy when appropriate [15].

*This recommendation has not been revised*.

9. In patients with personal breast cancer history, lymphadenopathy after vaccination should be interpreted considering the time since vaccination and overall nodal metastatic risk (cancer type, location, stage, etc.) [16]. For patients at low risk of axillary or supraclavicular nodal metastases in whom the lymphadenopathy is overwhelmingly more likely due to the vaccination than to the underlying neoplasm (considering time frame, pain, type, and location of cancer), a cautious management strategy without default follow-up imaging is appropriate. Short-interval follow-up imaging with ultrasonography (with at least a 12-week delay) may be performed in patients with higher risk of metastatic lymphadenopathy (e.g. breast cancer, head and neck cancer, upper extremity/trunk melanoma, or lymphoma). Node biopsy should be considered in the setting of high nodal metastatic risk when immediate histopathologic confirmation is necessary for timely patient management.

*This recommendation has not been revised*.

10. All complex or unclear cases (e.g. axillary lymphadenopathy ipsilateral to the cancer and the side of vaccination within 12 weeks after vaccination in patients with previous bilateral breast cancer; vaccinations performed on different sides) should follow a personalized management, considering the risk of malignant lymphadenopathy, opting for tissue sampling when appropriate after multidisciplinary team discussion.

*This recommendation has not been revised*.

## Conclusions

This update of the EUSOBI recommendations published in August 2021 aims at reducing unnecessary additional imaging and invasive procedures but, above all, at avoiding potential delays in breast cancer screening examinations. Future updates of these recommendations will be considered if the evolving boosting recommendations or the development and commercialization of new vaccines and of adapted versions of already approved ones will engender substantial changes in the clinical scenario.

## Data Availability

Not applicable.

## Abbreviations

BI-RADS: Breast Imaging Reporting and Data System
EUSOBI: European Society of Breast Imaging
SBI: Society of Breast Imaging
